# Modeling a Telemedicine Screening Program for Diabetic Retinopathy in Iran and Implementing a Pilot Project in Tehran Suburb

**DOI:** 10.1155/2019/2073679

**Published:** 2019-03-04

**Authors:** Sare Safi, Hamid Ahmadieh, Marzieh Katibeh, Mehdi Yaseri, Homayoun Nikkhah, Saeed Karimi, Ramin Nourinia, Ali Tivay, Mohammad Zareinejad, Mohsen Azarmina, Alireza Ramezani, Siamak Moradian, Mohammad Hossein Dehghan, Narsis Daftarian, Davood Abbasi, Afshin Eshghi Fallah, Bahareh Kheiri

**Affiliations:** ^1^Ophthalmic Research Center, Shahid Beheshti University of Medical Sciences, Tehran, Iran; ^2^Ophthalmic Epidemiology Research Center, Shahid Beheshti University of Medical Sciences, Tehran, Iran; ^3^Center for Global Health, Department of Public Health, Aarhus University, Aarhus, Denmark; ^4^Department of Epidemiology and Biostatistics, School of Public Health, Tehran University of Medical Sciences, Tehran, Iran; ^5^Department of Ophthalmology, Torfeh Medical Center, Shahid Beheshti University of Medical Sciences, Tehran, Iran; ^6^New Technologies Research Center, Amirkabir University of Technology, Tehran, Iran; ^7^Ocular Tissue Engineering Research Center, Shahid Beheshti University of Medical Sciences, Tehran, Iran; ^8^Islamshahr Branch, Iranian Diabetes Society, Islamshahr, Iran

## Abstract

**Purpose:**

To model a community-based telescreening program for diabetic retinopathy (DR) in Iran and to implement a pilot project at the Iranian Diabetes Society (IDS) branch in a Tehran suburb.

**Methods:**

In this mixed model study, a web application called the “Iranian Retinopathy Teleophthalmology Screening (IRTOS)” was launched. The educational course for DR screening was established for general practitioners (GPs). Registered patients in IDS branch were recalled for fundus photography; images were transferred to the reading center via IRTOS to be graded by GPs, and patients were informed about the results via mobile messaging. All images were independently reviewed by a retina specialist as the gold standard. Patients who required further assessment were referred to an eye hospital.

**Results:**

Overall, 604 subjects with diabetes were screened; of these, 50% required referral. The sensitivity and specificity for diagnosis of any stage of DR by trained GPs were 82.8% and 86.2%, respectively, in comparison to the gold standard. The corresponding values for detecting any stage of diabetic macular edema (DME) were 63.5% and 96.6%, respectively.

**Conclusions:**

Telescreening was an effective method for detecting DR in a Tehran suburb. This screening model demonstrated its capacity for promoting diabetic eye care services at the national level. However, the sensitivity for detecting DME needs to be improved by modifying the referral pathway and promoting the skill of GPs.

## 1. Introduction

Diabetes mellitus (DM) is a global epidemic and one of four priority noncommunicable diseases worldwide [1]. The prevalence of DM-related complications, such as diabetic retinopathy (DR), increased from 1990 to 2016, and DR is currently the leading cause of visual impairment (VI) and blindness in working age populations [2–5]. Among six World Health Organization (WHO) regions, the Eastern Mediterranean Region (EMR) demonstrated the highest growth rate in the prevalence of DM between 1980 and 2014 [1]. The burden of DM is high in Iran, rising by 35% among the Iranian adult population from 2005 to 2011; the number of individuals with DM is projected to be approximately 9.2 million by 2030 [6, 7]. Almost 25% and 30% of adults with DM are affected by DR in EMR and Iran, respectively [8–11].

Early detection and timely treatment is an effective approach to prevent approximately 90% of blindness and severe VI due to DR [12]. DR meets the Wilson and Jungner criteria for evaluating the needs of a screening program [13]. The WHO, International Council of Ophthalmology (ICO), clinical guidelines, and the National Program for Prevention and Control of Diabetes (NPPCD) have recommended regular eye examinations in patients with DM [14–17]. Despite these recommendations, population-based studies performed in Iran have reported a gap in regular eye examination in individuals with DM [10, 11]. One of these studies reported that 41.7% of individuals with DM had no history of ocular examination. Another study showed that less than one-fourth of individuals with DM underwent periodic eye examinations [10, 11]. A national assessment of DM and DR management systems using a WHO-recommended tool demonstrated the lack of network between DM and DR care services and limited screening coverage for DR in spite of sufficient and accessible technology and workforce [18].

Telemedicine is an innovative strategy to increase the screening rate by overcoming access barriers, promoting the network between care providers in primary and referral care settings, and improving patient awareness [16, 19–21]. Telescreening programs for DR have been successfully implemented in other countries [22–24]. Since 2003, the United Kingdom (UK) has operated the largest public screening program for DR, known as the English NHS Diabetic Eye Screening Program. The program's uptake was 82.8% in 2015-2016, resulting in the elimination of any certifiable blindness due to DR in the working age population [22]. Various successful programs have been designed and implemented in the United States [21, 24, 25]. Two published studies reported an increased screening rate for DR after implementation of telescreening program in primary health centers [21, 25]. Despite the advantages of this approach, a systematic telescreening DR program had not been launched in Iran before the present study.

To address the aforementioned gaps in the screening of DR in Iran, a community-based telemedicine screening program was modeled and implemented at the Islamshahr Branch of the Iranian Diabetes Society (IBIDS) in a Tehran suburb. This model was focused on involving the primary care setting to promote the awareness of both patients and DM care providers and to improve the network between DM and DR care services.

## 2. Materials and Methods

This mixed method study was conducted in three phases. Phases I and II were qualitative and focused on the designing of the screening process, web application, and implementing a training course. In phase III (the quantitative part), the screening model was implemented at the IBIDS. The study was approved by the Ethics Committee at Ophthalmic Research Center affiliated to Shahid Beheshti University of Medical Sciences, Tehran, Iran.

### 2.1. Protocol

#### 2.1.1. Phase I

The protocols, clinical pathways, and software used in the established programs, including the English NHS Diabetic Eye Screening Program [22], EyePACS (United States) [24], and Ophdiat® network (France) [23], were reviewed at several consensus group sessions of experts.

In the current model, all patients ≥12 years of age with DM were invited for screening. Patients were informed about the study and signed a consent form. For participants who aged less than 18 years, the consent form was signed by their parents.

Presenting visual acuity (PVA) was assessed. The measurement was repeated with pinhole if PVA was ≤20/40 (0.3 logMAR).

Three 45 degree mydriatic fundus images centered on the macula, and on the nasal and temporal retina, were captured from each eye. The pupil was dilated by instilling one mydriatic eye drop. Imaging was performed without pupil dilation if the patient had a history of increased intraocular pressure.

**Figure 1:**
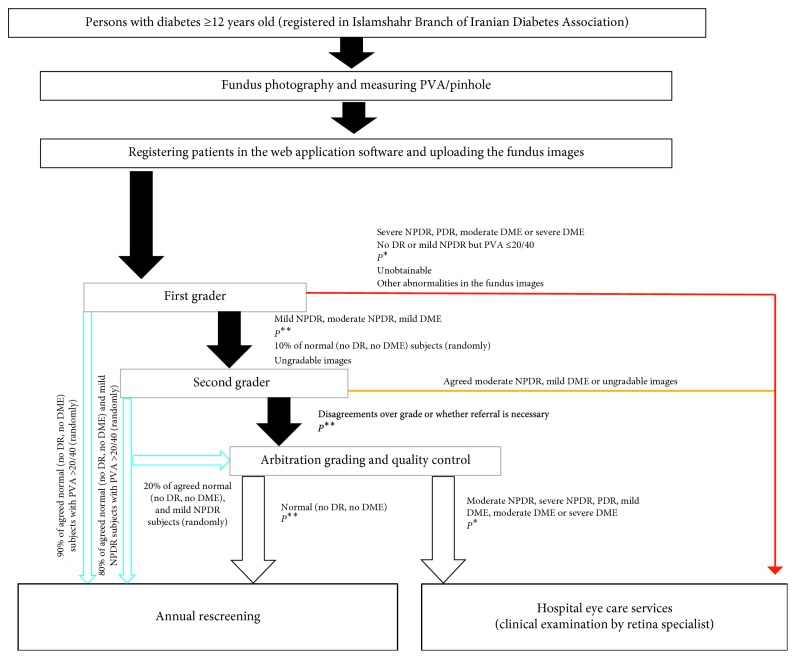
Patient care and referral pathway. DME: diabetic macular edema; DR: diabetic retinopathy; NPDR: nonproliferative diabetic retinopathy; PDR: proliferative diabetic retinopathy; MPC: macular photocoagulation; PRP: panretinal photocoagulation; PVA: presenting visual acuity. *P*^*∗*^: “evidence of PRP: active PDR” or “evidence of MPC: nonresolved DME”; *P*^*∗∗*^: “evidence of PRP:  regressed PDR” or “evidence of MPC: resolved DME.” Blue: annual rescreening; orange: nonurgent referral; red: urgent referral.

The International Clinical Classification System for Diabetic Retinopathy and Diabetic Macular Edema was chosen for grading the fundus images, with minor modifications. The patient care and referral pathway and the quality assurance process are shown in Figure 1. Patients who had severe nonproliferative DR (NPDR), proliferative DR (PDR), moderate or severe DME, and no DR or mild NPDR but PVA ≤20/40 (≥0.3 logMAR) were referred to the hospital urgently (in <4 weeks) for further evaluation. Fundus images that were graded as mild or moderate NPDR, and/or mild DME, and ungradable images were evaluated again by the second independent grader. In case of any disagreement, the fundus images were assessed by a retina specialist as an arbitrator to make the final decision. The patients with no DR and mild NPDR were recommended for the annual rescreening, and those with moderate NPDR and/or mild DME were referred to the hospital within <13 weeks (nonurgent referral).

A web application software was designed for storing and transferring the patient data and fundus images in collaboration with the New Technologies Research Center, Amirkabir University of Technology, Tehran, Iran. The application was launched in eight steps. In step one, the required demographic and clinical data were listed and were reviewed by experts in the field of ophthalmology, public eye care, diabetes, and computer science in at least 10 consensus sessions. In step two, the software and hardware infrastructures were set. A Ruby on Rails framework was provided as the output of this step to test, implement, and develop the system. In step three, the main structure and overall function of the application were designed. The schemes, along with the functional analysis, were collected as the output of step three. In step four, the initial graphic and the process details were designed. The overall structure of the program with complete details was the output of this stage. The coding was completed in step five, and its output was the initial version of the software application. Consequently, the turnover and safety of the software was tested and modified during step six. In step seven, the graphic and the style were finalized and, in the final step, the software was run on the hybrid server. Finally, a web application software called the “Iranian Retinopathy Teleophthalmology Screening (IRTOS)” was developed in Farsi language. It provided safe access by allocating an individual account for each of the users. The data also were encrypted before transferring for security reasons. Embedding of the referral care pathway (Figure 1) into the IRTOS modeling made the automatic classification of patients into three groups possible. These three groups consisted of urgent referral, nonurgent referral, and annual rescreen.

#### 2.1.2. Phase II

A 15-day upskilling course was established at the Ophthalmic Research Center affiliated to Shahid Beheshti University of Medical Sciences to increase the knowledge of general practitioners (GPs) about the screening of DR and to teach them how to interpret the fundus images of patients with DM. Eight academic retina specialists, a community medicine specialist, and a PhD student in the field of public eye care were involved in the training process.

Three learning methods, including theoretical, practical, and problem-based approaches, were used for designing the interactive sessions. The concepts of DR screening and the procedure of fundus photography were presented at first. Participants were trained for grading the fundus images via reviewing and discussing 300 color fundus photographs. Participants also observed clinical signs in the patients and practiced the screening tests in the eye clinic under the supervision of a retina specialist. International Council of Ophthalmology Guidelines for Diabetic Eye Care was translated to Farsi and introduced to participants as the instructional material [14]. Other sources included Clinical Practice Guidelines for Iranian Population and the Online Self-Directed Diabetic Retinopathy Grading Course affiliated to the University of Melbourne [17, 26]. The participants were assessed by both formative and summative evaluation methods during and at the end of the course. An official certificate was issued if the trainee successfully passed the assessment tests.

Furthermore, one of the IBIDS staff was instructed to capture fundus images. A schematic imaging protocol was provided to promote the photographer's skill. The trained staff was permitted to perform the photography for the screening program if the photographer's qualification was approved.

#### 2.1.3. Phase III

In the pilot phase, patients with DM and ≥12 years of age registered in the IBIDS were invited to participate in the screening program through a promotional meeting at the Islamshahr municipality conference hall. An informative brochure explaining the importance of screening for DR, the screening steps, and the contact number was distributed among the local community representatives.

According to the screening protocol described in phase I, PVA was measured using the high contrast Landolt “C.” The pupil was dilated using tropicamide eye drops (Mydrax 1%, Sina Darou, Iran), and fundus photography was performed using a fundus camera (CR2 Plus, Canon Inc., Tokyo, Japan). All data and fundus images were sent to the reading center at the Ophthalmic Research Center affiliated to Shahid Beheshti University of Medical Sciences via IRTOS. Two certified GPs graded the stage of DR and entered their interpretation into IRTOS. Patients who required further assessment were referred to a tertiary referral eye center (Torfeh Medical Center). Patients were informed of the screening result via a mobile text message. The IBIDS staff members were also updated through IRTOS. The assessment and required treatment records were entered into IRTOS. In subjects who declined the recommended clinical examination, a telephone interview was performed to identify the reason(s).

All images were independently reviewed by a senior retina specialist as the gold standard to evaluate the sensitivity and specificity of the GPs' grading in the pilot phase.

### 2.2. Statistical Analysis

Data were expressed as mean, standard deviation, median, range, and frequency. Sensitivity and specificity were calculated to evaluate the discriminant power of the system. To have a measure of precision, 95% confidence interval (CI) was calculated. All statistical analyses were performed using SPSS version 24.0 (IBM Corporation, Armonk, NY, USA) for Windows (Microsoft Corporation, Redmond, WA, USA).

## 3. Results

A total of 604 patients with DM (415 women, 189 men; mean age: 53 ± 14 years) were screened in phase III of the study from April to August 2018. Of the 604 subjects, 531 (91.6%) had Type 2 DM. The mean duration of DM was 9 ± 7 years (range, 1–36 years). A hemoglobin A1C level ≤7% was recorded in 35.1% of individuals, and 44.4% of the subjects were under treatment with oral antidiabetic medications. The demographic characteristics and medical data of the participants are summarized in Table 1.

Mean PVA in the worse eye was 0.26 ± 0.37* *logMAR, and 26.8% of the participants used glasses for distance vision. PVA ≤20/40 (≥0.3* *logMAR) was observed in 19.4% of participants.

The classification of DR stages was possible in 93.5% of subjects. Fundus photography could not be performed in 35 (5.8%) cases, and fundus images were not gradable in four (0.7%) patients. Cataract (*n*=13), posterior capsule opacification (*n*=4), pathologic myopia (*n*=3), pterygium (*n*=2), corneal opacity (*n*=2), and vitreous hemorrhage (*n*=1) were the primary causes of unsuccessful photography and low-quality images.

The sensitivity and specificity of the GPs for detecting any stage of DR were 82.8% (95% CI 79.51–85.57) and 86.2% (95% CI 83.77–88.31), respectively, compared with those of the retina specialist. The sensitivity of the GPs for detecting any stage of DME was 63.5% (95% CI 54.37–71.71), and the specificity was 96.6% (95% CI 95.47–97.41). The mean sensitivity and specificity of the GPs for detecting the ungradable images were 100% (95% CI 75.75–100) and 98.8% (95% CI 98.08–99.23), respectively.

One-half of the screened patients (*n*=302) did not need to be referred. These subjects were recommended to return to IBIDS for fundus photography after one year. Based on image interpretation, 40%, 13.7%, and 15.5% of the screened subjects had DR, DME, and sight-threatening DR, respectively (Table 2). Of 302 patients who required further evaluation, 229 (75.8%) complied with the recommendation and were visited by a retina specialist at the tertiary referral center (Torfeh Medical Center). The mean time between the screening event and the clinical examination was 27 ± 9 days (1–49 days).

Table 3 shows that 6.51% of individuals had moderate or severe DME associated with PVA better than 20/40 (<0.3 logMAR) in the worse eye.

In addition to detection of DR and DME, cataract was diagnosed in 24.5%, age-related macular degeneration in 13.5%, amblyopia in 2.2%, and branch retinal vein occlusion in 0.9% of patients who underwent clinical examination.

Fifty-five of 73 individuals who declined eye examination were interviewed. Factors affecting subjects' compliance included preference to be visited at a private center (41.8%), lack of awareness (34.5%), and transportation barriers (27.3%).

## 4. Discussion

In the current study, a community telescreening model for DR was developed and implemented at the IBIDS in a suburb of Tehran. A secure web-based telemedicine system called IRTOS was designed in local language and was successfully used by all users. The short-term training course for DR screening was established and approved by the Continuing Medical Education Center affiliated to Shahid Beheshti University of Medical Sciences and the Ministry of Health and Medical Education for the first time in Iran. A total of 604 patients with DM were screened in the pilot phase, in which the majority was women. Our study demonstrated that the quality of retinal images was appropriate in 93.5% of the screened patients. One-half of screened individuals were recommended to be followed through fundus photography in the next year at the primary care setting (IBIDS). Seventy-five percent of patients (*n*=229) who required further assessment were referred to the tertiary hospital center, of whom 21.4% required treatment.

In terms of sex distribution, most of our screened subjects were women, which was in accordance with the study by Jani et al. study, performed in the United States [21]. This can be explained by the increased access to the DR screening care facilities, which could overcome cost and transport barriers. The mean age of the individuals in our investigation was 53 ± 14 years, which was similar to two studies from the United States [21, 25].

The rates and causes of ungradable images were considered to be important issues by the American Telemedicine Association [27]. Our results demonstrated that >90% of fundus images had sufficient quality for grading, and cataract was the primary cause for deteriorating quality. Schulze-Döbold et al. and Zhang et al. demonstrated similar findings in France and China [23, 28]. Using the mydriatic photography method and the skill of photographer were the most important reasons to achieve the high rate of good-quality images [29]. Cavallerano et al. reported that, in the majority of patients with poor-quality images, ocular diseases required treatment. This finding is consistent with our findings [30]. As indicated in previous published studies, cataract and posterior capsular opacification were the major causes of low-quality images in the present study [31].

The sensitivity and specificity of GPs for identifying the ungradable images were high, which may be explained by the high number of sample images reviewed during the training course. In the present study, the trained GPs diagnosed any stage of DR with sensitivity of 82.8% and a specificity of 86.2% compared with the retina specialist. Previous research from Australia and Spain reported higher sensitivity and specificity for detecting DR by GPs and family physicians compared with ophthalmologists [32, 33]. However, in another report from Spain, Rodríguez Villa et al reported a sensitivity of 62% and specificity of 76% for grading of DR by GPs [34]. The sensitivity of GPs was 63.5% and the specificity was 96.6% for identifying any stage of DME, which were lower than the values reported by Askew et al. from Australia [35]. Considering the prediction of the lower sensitivity for DME detection due to lack of stereoscopic images by GPs, VA was added to the screening process. A better outcome can be achieved by improving quality assurance and continuous training, especially for DME detection. E-learning is an effective alternative strategy that provides an opportunity to improve the skill of GPs continuously. On the other hand, optical coherence tomography, along with fundus photography, was used for screening of DR in some studies; however, this remains controversial [36, 37].

Only one-half of the screened patients required further assessment, in which 31% were due to DR and DME. The referral rate was reported to be 9.3% to 31.2% in previous studies from different countries [21, 25]. Although the referral rate in our study was higher than reported values in the literature, it made follow-up of 50% of the individuals possible at the IBIDS as a primary health center. Hence, diagnosis and treatment resources could be allocated to patients with sight-threatening DR who were at a higher risk for blindness and severe VI [21].

More than two-thirds of patients adhered to recommendations for further assessment in our study. The mobile text message is recommended to increase the rate of adherence to the referral protocol [38–40]. This method was used for informing patients about the necessity and timing of clinical examination. The effect of mobile text message on the adherence rate will be evaluated in the future studies.

In our study, a table-top camera was used to capture the fundus photos. Smartphones and hand-held cameras have been utilized as the low cost, lightweight, and portable imaging modalities [41]. Validation studies have reported a wide range of sensitivity and specificity for detecting DR using these devices [41, 42]. A recently published review article reported the sensitivities of 64–93% and specificities of 72–100% for detecting DR by smartphone-based fundus photography [41]. Automated grading is also emerging for DR screening with the sensitivities and specificities of 87.0–95.2% and 49.6–68.8%, respectively [43]. Despite the advantages of these techniques, the image quality is a challenging issue [42].

## 5. Conclusions

A community-based telemedicine screening program for DR was modeled in a Tehran suburb. This screening model was implemented at IBIDS in Tehran. The current study focused on the integration of DM and eye care services, promoting the awareness of DM care providers and patients and applying modern technologies to improve DR care service delivery. Establishing web application software in the Farsi language and a short-term training course for screening of DR for GPs were the strengths of the study. Our findings demonstrated that this method of screening enabled the follow-up of at least 50% of patients with DM at a primary health center, and resources could be allocated to patients who required diagnostic and treatment interventions. The findings were presented to the Center for Non-Communicable Diseases Control, Ministry of Health and Medical Education, to promote diabetic eye care at the national level. In order to expand this program, the screening protocol should be modified to increase the validity parameters for detecting DR and DME. Future studies with larger sample size are needed to evaluate the modified screening model.

## Figures and Tables

**Table 1:** Demographic characteristics and medical data of the screened subjects.

Parameter	Level	Statistic	Value
Age (years)		Mean ± SD	53 ± 14
		Median (range)	56 (12–86)
	≤25	*N* (%)	37 (6.1)
	26–45	*N* (%)	110 (18.2)
	46–65	*N* (%)	366 (60.6)
	>66	*N* (%)	91 (15.1)

Sex	Female	*N* (%)	415 (68.7)
	Male	*N* (%)	189 (31.3)

Type of diabetes	Type 1	*N* (%)	49 (8.4)
	Type 2	*N* (%)	531 (91.6)

Duration of diabetes (years)		Mean ± SD	9 ± 7
		Median (range)	8 (1–36)
	<10	*N* (%)	397 (65.7)
	10–20	*N* (%)	165 (27.3)
	>20	*N* (%)	42 (7)

Hemoglobin A1C		Mean ± SD	7.82 ± 1.6
		Median (range)	7.6 (4.8–15)
	≤7	*N* (%)	212 (35.1)
	7–7/9	*N* (%)	116 (19.2)
	8–9	*N* (%)	164 (27.2)
	>9	*N* (%)	112 (18.5)

Current treatment	No treatment (improving life style)	*N* (%)	7 (1.2)
	Oral medication	*N* (%)	268 (44.4)
	Insulin	*N* (%)	120 (19.9)
	Combination therapy	*N* (%)	209 (34.6)

*N *=* *number; SD* *=* *standard deviation. Combination therapy = oral and insulin therapies.

**Table 2:** Results of the fundus image interpretations.

	*N* (%)
*Grading of DR*	
No DR	378 (60)
DR	226 (40)
** **Mild NPDR	42 (7.4)
** **Moderate NPDR	122 (21.6)
** **Severe NPDR	36 (6.4)
** **PDR	18 (3.2)
** **Evidence of PRP (regressed PDR)	5 (0.9)
** **Evidence of PRP (active PDR)	3 (0.5)
*Grading of DME*	
No DME	527 (86.3)
DME	77 (13.7)
** **Mild DME	24 (4.26)
** **Moderate DME	43 (7.6)
** **Severe DME	9 (1.6)
** **Evidence of MPC (nonresolved DME)	1 (0.2)
** **Evidence of MPC (resolved DME)	–

DR = diabetic retinopathy; DME = diabetic macular edema; *N* = number; MPC = macular photocoagulation; NPDR = nonproliferative diabetic retinopathy; PRP = panretinal photocoagulation.

**Table 3:** Classification of patients based on presenting visual acuity (PVA) and diabetic macular edema (DME).

Category	*N* (%)
1	403 (70.95)
2	112 (19.72)
3	37 (6.51)
4	16 (2.82)

*N* = number. 1 = PVA with pinhole >20/40 and no DME or mild DME; 2 = PVA with pinhole ≤20/40 and no DME or mild DME; 3 = PVA with pinhole >20/40 and moderate or severe DME; 4 = PVA with pinhole ≤20/40 and moderate or severe DME.

## Data Availability

The data used to support the findings of this study are available from the corresponding author upon request.
